# Uncovering lupus nephritis-specific genes and the potential of TNFRSF17-targeted immunotherapy: a high-throughput sequencing study

**DOI:** 10.3389/fimmu.2024.1303611

**Published:** 2024-02-19

**Authors:** Xiaojuan Zou, Mingyue Yang, Zhuang Ye, Tie Li, Zhenyu Jiang, Ying Xia, Shenghai Tan, Yu Long, Xiaosong Wang

**Affiliations:** ^1^ Department of Rheumatology and Immunology, The First Hospital of Jilin University, Changchun, China; ^2^ Laboratory for Tumor Immunology, The First Hospital of Jilin University, Changchun, China; ^3^ Department of Surgical Intensive Care Unit (SICU), The First Affiliated Hospital of Xi’an Jiaotong University, Xi’an, China

**Keywords:** lupus nephritis, high-throughput sequencing analysis, microarray analysis, TNFRSF17, IBI379

## Abstract

**Introduction:**

Lupus nephritis (LN) is a severe manifestation of systemic lupus erythematosus (SLE). This study aimed to identify LN specific-genes and potential therapeutic targets.

**Methods:**

We performed high-throughput transcriptome sequencing on peripheral blood mononuclear cells (PBMCs) from LN patients. Healthy individuals and SLE patients without LN were used as controls. To validate the sequencing results, qRT-PCR was performed for 5 upregulated and 5 downregulated genes. Furthermore, the effect of the TNFRSF17-targeting drug IBI379 on patient plasma cells and B cells was evaluated by flow cytometry.

**Results:**

Our analysis identified 1493 and 205 differential genes in the LN group compared to the control and SLE without LN groups respectively, with 70 genes common to both sets, marking them as LN-specific. These LN-specific genes were significantly enriched in the ‘regulation of biological quality’ GO term and the cell cycle pathway. Notably, several genes including TNFRSF17 were significantly overexpressed in the kidneys of both LN patients and NZB/W mice. TNFRSF17 levels correlated positively with urinary protein levels, and negatively with complement C3 and C4 levels in LN patients. The TNFRSF17-targeting drug IBI379 effectively induced apoptosis in patient plasma cells without significantly affecting B cells.

**Discussion:**

Our findings suggest that TNFRSF17 could serve as a potential therapeutic target for LN. Moreover, IBI379 is presented as a promising treatment option for LN.

## 1 Introduction

Systemic lupus erythematosus (SLE) is an autoimmune disease that manifests as a spectrum of clinical presentations due to defects at various points in the immune cascade (1–3). Approximately 50% of SLE patients experience kidney involvement, with lupus nephritis (LN) being a significant risk factor for morbidity and mortality. Despite the availability of anti-inflammatory and immunosuppressive treatments, many patients still develop chronic kidney disease (CKD) or end-stage renal disease (ESRD) (4, 5). Despite the rapid pace of new drug discovery, most clinical trials of well-designed treatments failed in LN due to poor efficacy or significant side effects. Consequently, there exists an imperative necessity to comprehensively comprehend the intricate immune processes that underlie LN.

One of the important advances in the treatment of LN in recent years is the use of B-cell-targeted therapy. B-cell activating factor (BAFF) and its homologue, a proliferation-inducing ligand (APRIL), are TNF-like cytokines that support the survival and differentiation of B cells at different stages of development. The BAFF family receptors (BAFFR), transmembrane activator calcium modulator and cyclophilin ligand interactor (TACI), and TNF receptor superfamily member 17 (TNFRSF17), are three surface receptors for BAFF and APRIL. The BAFF inhibitor, belimumab, is the first biologic drug approved for SLE treatment (6–8), which improves renal outcomes in active LN patients when used in combination with standard therapy in a randomized controlled trial (RCT) study (9). However, its efficacy was slow and mild, which is inadequate for acute severe LN patients. Telitacicept, approved in China for active SLE treatment, lacks sufficient evidence of action through APRIL, with overexpression in mice showing mild immune abnormalities and insufficient autoimmune disease features (10); its use in LN treatment is linked with immunosuppression and increased infection risk (11). Consequently, there is an urgent requirement for a treatment that is both more efficacious and safer for LN.

Omic techniques, such as transcriptomic techniques, have become essential tools for exploring the molecular processes involved in the development of diverse diseases from an academic perspective (12). Transcriptome studies were employed to identify key pathogenic drivers and characterize the genetic pathways involved in LN (13, 14). However, the identification of effective therapeutic targets for LN remains elusive. In this study, we hypothesized that the mRNA expression profile of PBMCs in LN patients differs from that of SLE without LN patients and healthy controls. To explore the unique pathogenic genes associated with LN, we performed transcriptomic sequencing on PBMCs from LN patients, SLE without LN patients, and healthy controls. By comparing the microarray results of LN patient kidneys, we identified key pathogenic genes for further investigation.

## 2 Methods

### 2.1 Participants

All samples from patients were obtained from the Department of Rheumatology of the First Hospital of Jilin University (Changchun, China). The SLE patients included in this study met at least four of the eleven criteria for SLE as revised by the American College of Rheumatology (ACR) (15), and LN was defined as 24-hour urinary protein of more than 0.5g. Patients with a current or recent infection were excluded from the study. The severity of the disease was assessed using the SLE disease activity index 2000 (SLEDAI-2K) (16). Healthy controls were recruited as volunteers with no history of autoimmune disease or immunosuppressive therapy, and were frequency-matched with the patients for age and sex. All participants were of Han Chinese ethnicity. A total of 49 LN patients, 46 SLE patients without LN, and 38 healthy controls were included in the study. Ten samples from each group were used for sequencing, while the remaining samples were used for qRT-PCR validation and cell culture. Peripheral blood mononuclear cells (PBMCs) were isolated from whole blood using density gradient centrifugation (Lymphopre, Axis-Shield, Scotland).

### 2.2 RNA isolation and sequencing

RNA isolation and sequencing were performed as previously described by Yang et al. (17). Briefly, after cluster generation, library preparations were sequenced on an Illumina HiSeq X Ten, and 150 bp paired-end reads were generated.

### 2.3 Sequencing data analysis

Sequencing data analysis were performed as previously described by Yang et al. (17). Briefly, Raw sequencing data in fastq format were initially processed using in-house perl scripts. Ensembl database (Homo_sapiens. GRCh38.94) were used, and paired-end clean reads were aligned to the reference genome using HISAT2 v2.0.4 (18). The sequencing data analysis software used for difference analysis is edgeR (3.0.8). The *p*-values were adjusted using Benjamini and Hochberg’s approach to control the false discovery rate (FDR), and genes with a corrected *p*-value < 0.05 were identified as differentially expressed (DE). GO enrichment analysis and KEGG pathway analysis were performed as previously described (19, 20). GO terms and KEGG pathways with *p* < 0.05 were considered significantly enriched.

### 2.4 Quantitative reverse transcription-polymerase chain reaction

Total RNA was extracted from cells using the TRIzol reagent (Invitrogen, Carlsbad, California, USA) and stored at -80°C (5 × 10^6^ cells/mL). qRT-PCR was conducted as previously described (21).

### 2.5 Microarray analysis

Gene expression profiles were analyzed using microarray data obtained from the GEO database (accession numbers: GSE32583 for NZB/W mice kidney tissues and GSE32591 for human glomerular and renal tubular tissues) (22). Kidney tissues were from the whole kidneys of NZB/W mice (accession number: GSE32583) or human glomerular and renal tubular tissues (accession number: GSE32591). Mice were grouped as previously described (23), and details can be found on the GEO website (https://www.ncbi.nlm.nih.gov/geo/geo2r/?acc=GSE32583). Human kidney tissue samples were obtained from renal biopsies (22), and grouping was performed as previously described (23). Details are provided at the GEO website (https://www.ncbi.nlm.nih.gov/geo/geo2r/?acc=GSE32591). Differential gene analysis was conducted using a *p*-value threshold of less than 0.05 and a fold-change in expression of more than 1.2.

### 2.6 Immunohistochemistry staining

Immunohistochemistry staining was performed according to previously described methods (24), using the percentage of TNFRSF17^+^ area were determined using Image J software (25). Kidney tissues were fixed in a 10% neutral formalin solution, followed by embedding in paraffin and subsequent dewaxing and slicing. An immunohistochemistry assay was then performed. Firstly, the tissues were incubated with an oxidase blocking solution at room temperature. Subsequently, animal serum was added, followed by overnight incubation with a primary antibody against TNFRSF17 (ab199264; Abcam, USA). Visualization was achieved by adding a secondary antibody and hematoxylin. The resulting IHC staining was evaluated using ImageJ software (National Institutes of Health, Bethesda, Maryland). TNFRSF17 immunoreactivity was quantified in three randomly selected representative slide areas (×400 magnification) using a light microscope. The percentage of TNFRSF17-positive area out of the total area was calculated (23, 24, 26–28).

### 2.7 Cell culture and flow cytometry analysis

RPMI 1640 (Corning, NY, USA) medium (containing 100 units/mL of penicillin and 100 μg/mL of streptomycin) with 10% patients’ plasma were used to culture PBMCs in a 12-well plate. The wells were divided into the IBI379 (0.1 μg/mL in 0.9% sodium chloride solution) group and the control group (equal volume of 0.9% sodium chloride solution). Three wells for each group. IBI379 was generously provided by Innovent Company (Suzhou, Jiangsu, China). After 24 hours of incubation, the cells were collected and stained with BD Horizon™ BV711 Mouse Anti-Human CD19 (563036, BD, USA), BD Pharmingen™ PerCP-Cy™5.5 Mouse Anti-Human CD38 (551400, BD, USA), and BD Pharmingen™ PE-Cy™7 Mouse Anti-Human CD20 (560735, BD, USA). BD Horizon™ BV711 Mouse IgG1, κ Isotype Control (563044; BD, USA), BD Pharmingen™ PerCP-Cy™5.5 Mouse IgG1 κ Isotype Control (550795, BD, USA), and BD Pharmingen™ PE-Cy™7 Mouse IgG2b, κ Isotype Control (560542, BD, USA) were used for the controls. The detection of apoptosis was conducted using Annexin V-FITC/PI (CA1020, Solarbio, China), as per the instructions provided by the manufacturer. The procedure involved the addition of 5μL Annexin V-FITC to the cell tube, followed by gentle mixing and incubation at room temperature for 10 minutes, ensuring avoidance of light exposure. Subsequently, 5μL PI was added and the mixture was again incubated at room temperature, away from light, for a duration of 5 minutes. The mixture was then supplemented with 500μL of PBS, mixed gently, and subjected to flow cytometry. The analysis of the flow cytometry data was carried out using a Fortessa flow cytometer (BD, USA). 1×10^5^ events were collected for each sample.

### 2.8 Statistical analysis

GraphPad Prism 9.0 (GraphPad Software, San Diego CA, USA) was used for statistical analysis and statistical graph rendering. An unsupervised heatmap was generated using all data between the maximum (red) and minimum (blue) values for each gene to compare different matrices. The Wilcoxon signed-rank test for paired samples and Mann-Whitney *U* test for unpaired samples were applied. A *p*-value less than 0.05 was considered statistically significant.

## 3 Results

### 3.1 Clinical characteristics of the sequencing subjects

To investigate the pathogenesis of LN, we performed second-generation sequencing on 10 LN patients, 10 SLE without LN patients, and 10 healthy individuals. **Supplementary Table 1** summarizes the basic features and clinical information of the sequencing subjects. The median age of the sequencing LN patients was 39.5 years (range from 15 to 54), the median age of the SLE patients without LN was 32.5 years (range from 15 to 67), and the median age of the healthy controls was 31 years (range from 24 to 37). The SLEDAI score, anti-dsDNA antibody titer, 24-hour urine protein quantification, white blood cells, and neutrophils were significantly higher in the LN group than in the SLE without LN group (*p* < 0.05), while there were no significant differences in gender and age among the three groups. There were no significant differences in complement C3, complement C4, blood urea nitrogen, creatinine, and procalcitonin levels between the LN group and the SLE without LN group.

### 3.2 Identification and classification of differentially expressed genes

**Figure 1:**
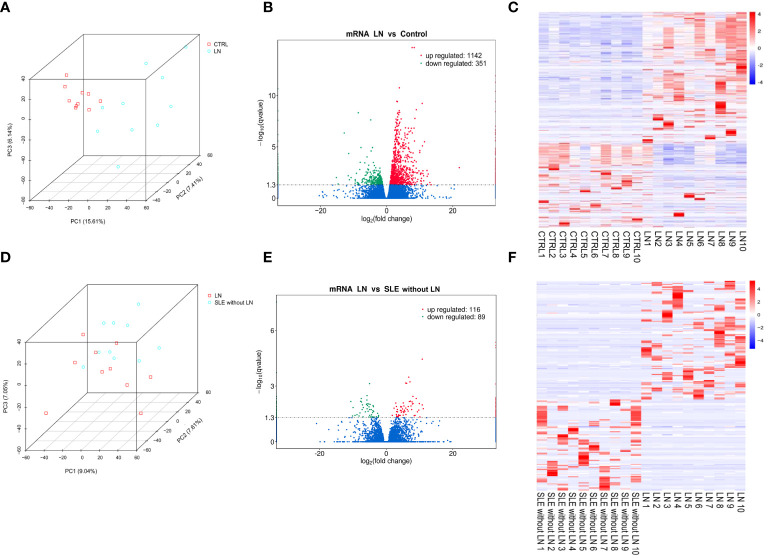
Evaluation of differentially expressed genes between groups. **(A)** Principal component analysis (PCA) plot of sequencing samples from the lupus nephritis (LN) group and control group, showing contribution ratios. **(B)** Volcano plot of genes differentially expressed between the LN group and control group, with each point representing a detectable gene in both groups. **(C)** Cluster of 1493 genes significantly different between the LN group and control group. **(D)** PCA plot of samples from the LN group and systemic lupus erythematosus (SLE) without LN group, showing contribution ratios. **(E)** Volcano plot of genes differentially expressed between the LN group and SLE without LN group, with each point representing a detectable gene in both groups. **(F)** Cluster of 205 genes significantly different between the LN group and SLE without LN group. Corrected-*p* < 0.05.

Principal component analysis (PCA) was employed to assess the clustering properties of the sequencing samples between the control group and the LN group (**Figure 1A**). In the PCA plot, each point represents a sample, and the position of the point reflects the sample’s scores on the principal components (29). We observed a clear clustering trend in the distribution of samples along the first principal component (PC1), second principal component (PC2), and third principal component (PC3). This suggests significant differences at the transcriptomic level between the control and LN groups, possibly associated with the disease state. As shown in **Figure 1B**, the volcano plot of genes differentially expressed between the LN group and the control group revealed 1493 differentially expressed mRNAs, including 1142 significantly upregulated genes and 351 significantly downregulated genes. The heatmap analysis (**Figure 1C**) showed significant differences in gene expression between the LN group and the control group in each sample. The specific information of the top 20 upregulated and downregulated genes in the LN group is shown in **Supplementary Tables 2**, **3**, respectively.

PCA was also used to evaluate the clustering properties of the sequencing samples between the LN group and the SLE without LN group (**Figure 1D**). Although there was some overlap, the two groups could be distinguished overall. There were 205 differentially expressed genes (**Figure 1E**), including 116 significantly upregulated genes and 89 significantly downregulated genes. The heatmap analysis (**Figure 1F**) showed significant differences in gene expression between the LN group and the SLE without LN group in each sample. The specific information of the top 20 upregulated and downregulated genes in the LN group is shown in **Supplementary Tables 4**, **5**, respectively.

### 3.3 Identification and classification of 70 genes specifically expressed in LN group

**Figure 2:**
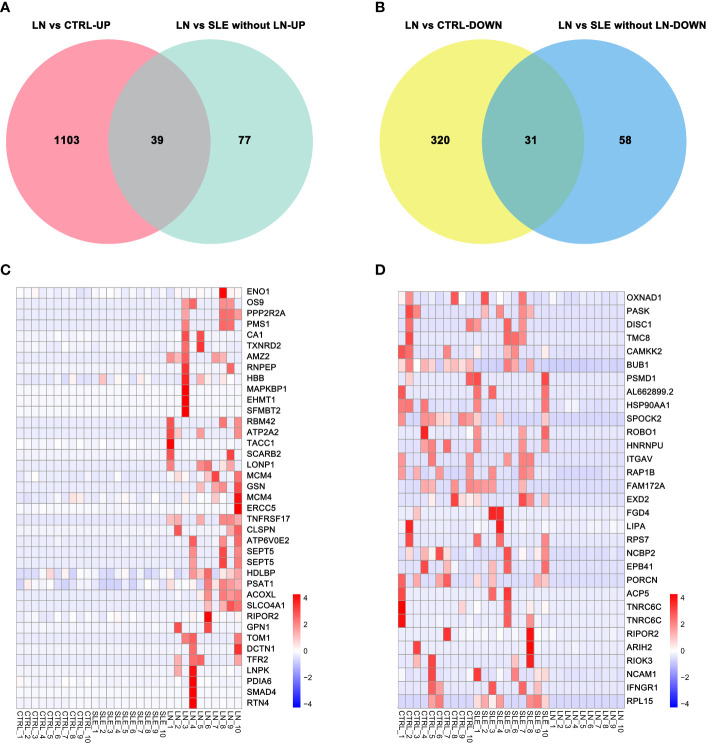
Differential genes specifically expressed in the LN group. **(A)** Venn diagram of upregulated genes. **(B)** Venn diagram of downregulated genes. **(C)** Cluster of upregulated genes in the LN group. **(D)** Cluster of downregulated genes in the LN group. Corrected-*p* < 0.05.

A Venn diagram analysis was performed to identify the specifically expressed genes in the LN group. We found that 70 genes were commonly expressed in the 205 differentially expressed genes between the LN group and the SLE without LN group, as well as the 1493 differentially expressed genes between the LN group and the control group. Among these 70 genes, 39 were upregulated (**Figure 2A**) and 31 were downregulated (**Figure 2B**). Heatmap analysis revealed the expression patterns of the 39 upregulated genes (**Figure 2C**) and the 31 downregulated genes (**Figure 2D**).

### 3.4 GO enrichment analysis

**Figure 3:**
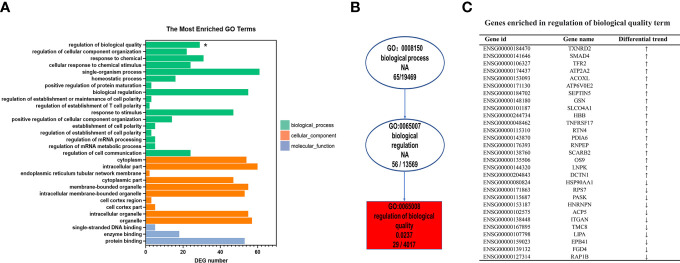
Gene Ontology (GO) enrichment analysis of differentially expressed genes specific to lupus nephritis (LN). **(A)** The top 30 GO terms were enriched as biochemical processes (BP), cellular components (CC), or molecular function (MF). **(B)** Directed acyclic graph (DAG) of the GO terms related with ‘Regulation of biological quality’. **(C)** List of mRNA genes enriched in the ‘Regulation of biological quality’term. *p < 0.05.

To further explore the biological functions of the differentially expressed genes in the LN group, we performed GO analysis based on the GO annotation terms. The enriched GO terms were classified into biological process (BP), cellular component (CC), and molecular function (MF) (**Figure 3A**). The ‘regulation of biological quality’ term was significantly upregulated (*p* < 0.05). Biological quality refers to measurable attributes of an organism or its parts, such as size, mass, shape, color, etc. ‘Regulation of biological quality’ encompasses any process that modulates a qualitative or quantitative trait of a biological quality. Furthermore, the directed acyclic graph revealed that the ‘regulation of biological quality’ term was under the biological regulation term, which belong to the biological process (**Figure 3B**). As shown in **Figure 3C**, 18 upregulated genes and 11 downregulated genes were enriched in the ‘regulation of biological quality’ term. For example, upregulated HBB, TNFRSF17, and SCARB2 are involved in this term.

### 3.5 KEGG pathway enrichment analysis

**Figure 4:**
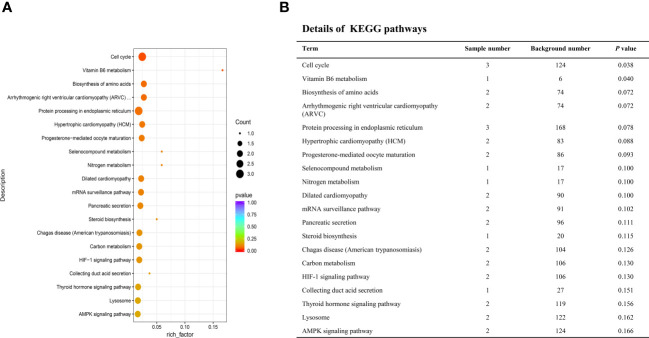
Kyoto encyclopedia of genes and genomes (KEGG) enrichment analyses of the deferentially expressed genes specific to LN. **(A)** Top 20 enriched pathways. The size of each circle represents the number of enriched genes, and different colors represent different q-values. **(B)** Detailed information of the top 20 enriched pathways.

We performed KEGG pathway enrichment analysis to investigate the biological pathways associated with the differentially expressed genes of the LN group (**Figure 4**). The analysis revealed that these genes were highly enriched in several pathways, including Cell cycle (SMAD4, BUB1, MCM4), Vitamin B6 metabolism (PSAT1), Biosynthesis of amino acids (ENO1, PSAT1), Protein processing in endoplasmic reticulum (HSP90AA1, PDIA6, OS9), mRNA surveillance pathway (PPP2R2A, NCBP2), and Lysosome (SCARB2, LIPA) among others.

### 3.6 Validation of 10 LN-specific mRNAs using qRT-PCR

**Supplementary Table 6** showed the clinical characteristics of the qRT-PCR subjects (Seven LN samples among them were used for cell culture). The LN group had significantly higher SLEDAI scores, blood urea nitrogen, 24-hour urine protein quantification, creatinine, and procalciton in compared to the SLE without LN group (*p* < 0.05). Additionally, the LN group had lower complement C3 levels compared to the SLE without LN group *(p* < 0.05). There were no significant differences in age or gender among the three groups. There were also no differences in white blood cell count, neutrophil count, or anti-dsDNA levels between the LN group and the SLE without LN group.

**Figure 5:**
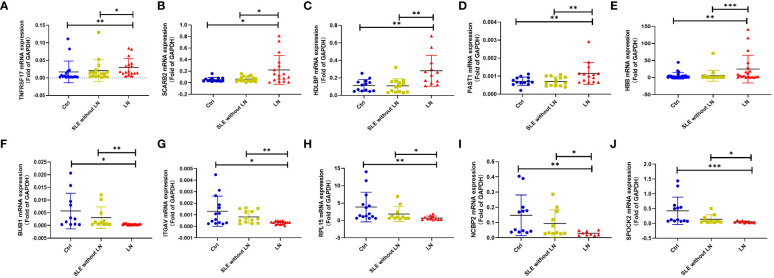
Validation of some differential expression genes specifically expressed in the LN group using quantitative real-time PCR (qRT-PCR). **(A–E)**. qRT-PCR validation of upregulated genes in the LN group compared to the control group or SLE without LN group. **(F–J)**. qRT-PCR validation of downregulated genes in the LN group compared to the control group or SLE without LN group (Ctrl n=28, SLE without LN n=36, LN n=32). *p < 0.05, **p < 0.01, and ***p < 0.001.

To validate the sequencing results, we performed qRT-PCR on 5 upregulated genes (TNFRSF17, SCARB2, HDLBP, PSAT1, HBB) and 5 downregulated genes (BUB1, ITGAV, RPL15, NCBP2, SPOCK2) (see **Supplementary Table 7** for primer information). The qRT-PCR results were consistent with the high-throughput sequencing results (**Figure 5**). Therefore, these sequencing results were validated successfully.

### 3.7 Gene expression in the kidneys

**Figure 6:**
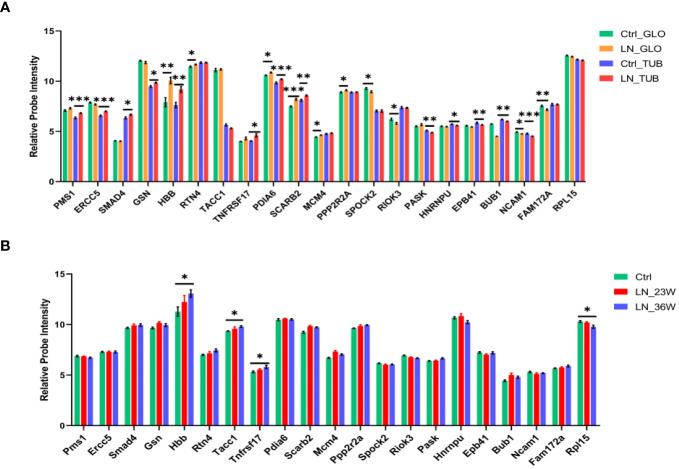
Expression levels of LN-specific genes in kidneys based on microarray analysis. **(A)** Histogram showing the microarray data in the kidneys from LN patients and healthy controls (Ctrl n=15; LN n=32). Glomerulus (GLO), renal tubular (TUB). **(B)** Histogram showing the microarray data in the kidneys from NZB/W mice (Ctrl n=8; LN_23w n=6; LN_36w n=10). The *p*-values are indicated as *p < 0.05, **p < 0.01, and ***p < 0.001.

To investigate the expression of the 70 LN-specific genes in the kidney, we conducted an analysis of microarray data obtained from kidney samples. **Figure 6A** illustrates the comparison of gene expression levels between the LN group and the healthy control group. Our results revealed a significant upregulation of HBB, RTN4, PDIA6, SCARB2, MCM4, and PPP2R2A in the glomeruli of the LN group. Conversely, SPOCK2, RIOK3, NCAM1, and FAM172A exhibited decreased expression in the glomeruli of the LN group. Analysis of the renal tubules in LN patients and healthy controls revealed significant differences in the upregulated genes PMS1, ERCC5, SMAD4, GSN, HBB, TNFRSF17, PDIA6, and SCARB2. Compared to healthy controls, LN patients had decreased expression of PASK, HNRNPU, EPB41, BUB1 and NCAM1 in the renal tubules (*p* < 0.05). These results provide valuable insights into the differential expression patterns of LN-specific genes in the kidney, elucidating potential molecular pathways that may contribute to the etiology of LN.

We analized the expression of the 70 differentially expressed genes in the kidney tissue of NZB/W mice (**Figure 6B**). Renal changes occur and progress with the increasing age of the NZB/W mice. Therefore, the 16-week mice serve as the normal control group, the 23-week mice represent the early-stage group of nephritis, and the 36-week mice represent the mid-late stage group of nephritis. The results indicate an increasing trend in the expression level of TNFRSF17 during the early stage of the disease and a significant upregulation in the mid-late stage group compared to the control group. In addition, results showed significant differences in the upregulated genes (Hbb and Tacc1) and the downregulated gene Rpl15 between the 36-week LN group and the control group. Therefore, TNFRSF17 and HBB were found to be statistically significant factors in the analysis of human and mouse kidney tissue.

To investigate the expression of these differentially expressed genes in resident lymphocytes versus kidney cells, we performed an analysis of single-cell RNA sequencing data obtained from human kidney (**Supplementary Figure 1**) and mouse kidney (**Supplementary Figure 2**). Our results revealed that HBB was predominantly expressed in erythroid lineage cells, TNFRSF17 was mainly expressed in B cells, SCARB2 showed primary expression in endothelial cells and macrophages, PPP2R2A exhibited primary expression in endothelial cells, and BUB1 was primarily expressed in proliferating B cells. To further confirm the expression of these genes in lymphocytes, we analyzed single-cell RNA sequencing data obtained from human peripheral blood mononuclear cells (PBMCs) (**Supplementary Figure 3**). Our findings demonstrated that HBB was mainly expressed in erythroblasts, TNFRSF17 showed primary expression in class-switched memory B cells, SCARB2 exhibited primary expression in inflammatory macrophages, PPP2R2A was primarily expressed in CD4+ T cells, and BUB1 showed primary expression in megakaryocytes.

### 3.8 The Expression of TNFRSF17 and correlation analysis

**Figure 7:**
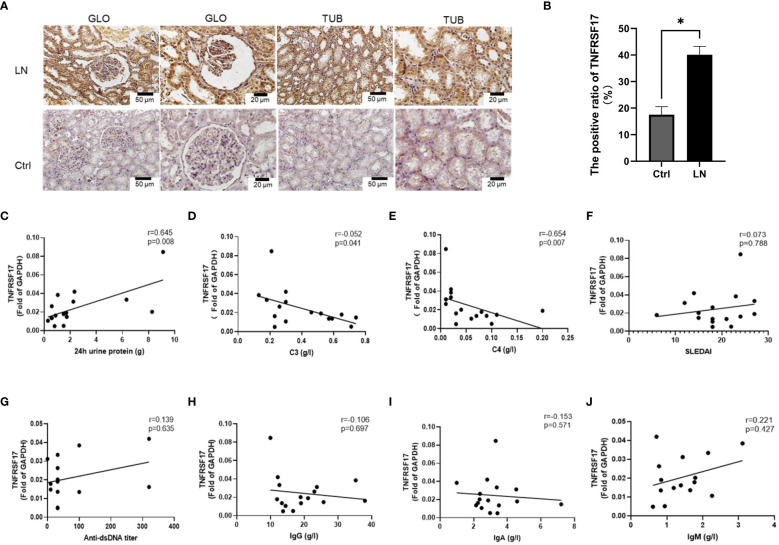
Protein levels of TNFRSF17 in the kidneys of LN patients. **(A)** Immunohistochemical staining of TNFRSF17 in the kidneys of LN patients and healthy controls. Glomerulus (GLO), renal tubular (TUB). **(B)** The percentage of TNFRSF17^+^ area in kidney was evaluated (n=4, *p* < 0.05) using ImageJ software. **(C-J)**. Correlation analysis between TNFRSF17 levels and clinical indicators.

The upregulation of the mRNA levels of TNFRSF17 was confirmed by qRT-PCR analysis of PBMCs from LN patients (**Figure 5A**). To further observe the protein levels of TNFRSF17 in human kidney tissue, we performed immunohistochemical staining and found that TNFRSF17 expression was significantly higher in LN patients than in healthy controls (**Figures 7A, B**). The clinical information about the immunohistochemistry samples, inclusive of pathological type (as per The 2003 ISN/RPS LN classification system (30) and the National Institutes of Health LN activity/chronicity scores (AI/CI), is enumerated in **Supplementary Table 8**. We undertook an exploration into the potential correlation between TNFRSF17 levels and the LN renal AI/CI score. However, as depicted in **Supplementary Figure 4**, the current findings do not provide evidence for a positive correlation between TNFRSF17 levels and the LN renal AI/CI score.

C3, C4, and related complement proteins are activated in SLE patients and deposited in inflammatory tissues, leading to a decrease in circulating complement levels that are negatively correlated with disease activity (31, 32). Treatment can increase complement levels, while complement activation during disease relapse can lead to a decrease in circulating complement levels again (33). Urinary protein quantification is an important indicator for monitoring the activity of kidney disease in LN patients (34). Correlation analysis between the mRNA levels of TNFRSF17 and clinical indicators in the LN group showed a positive correlation between TNFRSF17 and 24-hour urine protein quantification and a negative correlation with complement C3 and C4, which were statistically significant (**Figures 7C–E**). However, there was no statistically significant correlation between TNFRSF17 and SLEDAI, anti-dsDNA antibody titer, IgA, IgM, or IgG (**Figures 7F–J**).

### 3.9 Efficacy study of IBI379 in killing TNFRSF17^+^ plasma cells

**Figure 8:**
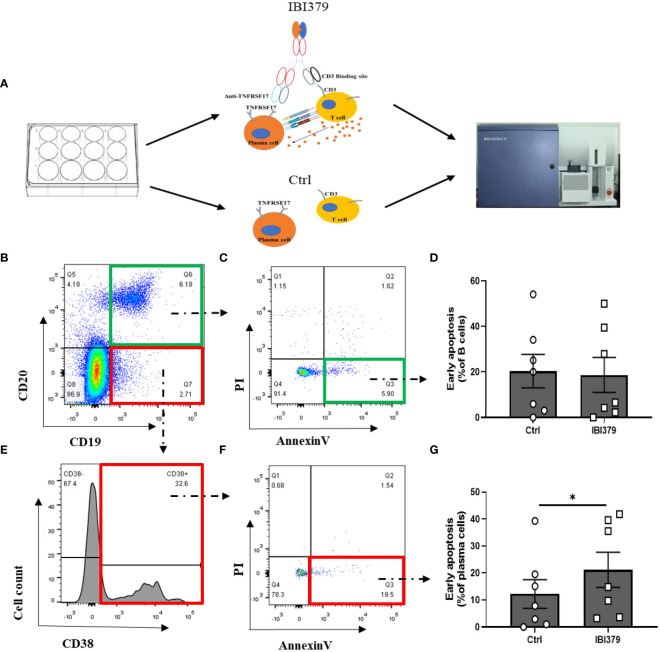
Flow cytometry analysis of CD19^+^CD20^+^ B cells and CD19^+^CD20-CD38^+^ plasma cells in peripheral blood mononuclear cells (PBMCs) of lupus nephritis (LN) patients after treatment with IBI379. **(A)** Workflow shows the experimental strategied and analysis in this study. **(B)** Representative flow cytometry plots of CD19^+^CD20^+^ B cells. **(C)** Annexin V/PI apoptosis assay of B cells. **(D)** Comparison of Annexin V/PI apoptosis assay results between control and treatment groups for B cells. **(E)** Representative flow cytometry plots of CD19^+^CD20^-^CD38^+^ plasma cells. **(F)** Annexin V/PI apoptosis assay of plasma cells. **(G)** Comparison of Annexin V/PI apoptosis assay results between control and treatment groups for plasma cells. The *p*-values are indicated as *p < 0.05.

We found that TNFRSF17 was highly expressed in both PBMCs and kidney tissue, but the effectiveness of TNFRSF17 as a therapeutic target remained unclear. As shown in **Figure 8A**, IBI379 is a construct consisting of three chains: a standard anti-TNFRSF17 heavy chain, a standard anti-TNFRSF17 light chain, and an anti-CD3-ScFv-Fc fusion chain (35). CD3 molecules deliver the first activation signal to T cells, and previous studies showed that IBI379 can effectively target TNFRSF17 and CD3, and inducing T cell activation, proliferation, and clearance of TNFRSF17^+^ plasma cells in multiple myeloma (MM) patients (35). We added IBI379 to the PBMCs of LN patients, cultured for 24 hours, and then detected the apoptosis of B cells and plasma cells. As shown in **Figures 8B-G**, results showed that there was no statistically significant difference in the effect of IBI379 on CD19/CD20 double-positive B cells compared to the control group (*p* > 0.05, **Figure 8D**), but IBI379 effectively kill CD19^+^CD20^-^CD38^+^ plasma cells in LN patients (*p* = 0.0328, **Figure 8G**).

## 4 Discussion

Due to the urgent need for efficient and low-toxicity drugs for LN, we performed high-throughput transcriptome sequencing on PBMCs from LN patients, analyzed differential genes, and identified 70 LN-specific differential genes. qRT-PCR validation of 5 upregulated genes and 5 downregulated genes showed consistent results with the sequencing results. These genes were enriched in the GO term of ‘regulation of biological quality’ and the cell cycle pathway. Additionally, through the comparison of microarray findings on these 70 genes in kidney tissue, we thoroughly examine the gene expression and potential pathways implicated in the development of LN, thereby proposing novel therapeutic targets for LN management. As far as we know, these differentially expressed genes unique to LN patients have not been reported in the microarray or qRT-PCR studies (36–43).

Firstly, SCARB2 was significantly elevated in PBMCs, glomeruli, and renal tubules of LN patients. Similar to the single-cell sequencing results we analyzed, people reported that SCARB2 was significantly upregulated in monocyte-derived macrophages in the kidney of LN patients (44, 45). Guo et al. found that SCARB2 was highly expressed in human plasmacytoid dendritic cells (pDC) and could regulate type I interferon production by mediating endosomal translocation of TLR9 and nuclear translocation of IFN regulatory factor 7 (46). IFN levels were elevated in LN patients (47). Type I interferon may break immune tolerance in SLE patients by activating dendritic cells and CD8 T cells (48). Therefore, SCARB2 may be an effective therapeutic target for LN, inhibiting SCARB2 can reduce the production of type I interferon.

Secondly, we observed a significant downregulation of BUB1 (Budding uninhibited by benzimidazoles-1) in PBMCs and renal tissue of LN patients. BUB1 encodes a serine/threonine protein kinase that plays a central role in mitosis. Overexpression of BUB1 was showed to promote tumor cell proliferation, migration, invasion, and reduce apoptosis (49–52). Nyati et al. found that BUB1 promotes the formation of the TGFBRI/II receptor complex and regulates downstream signaling in the Smad pathway, which is an important component of TGFβ signaling transduction (53). The differentiation of Treg cells depends mainly on the binding of TGFβ to the TGFBRI/II receptor complex on the surface of naïve T cells, which activates Smads (R-Smad) and forms a complex with Smad4 that translocates to the nucleus and induces forkhead box p3 (Foxp3) gene expression (54). Therefore, the downregulation of BUB1 in LN may inhibit the differentiation of Treg cells through the TGFβ/Smads pathway, leading to immune tolerance dysfunction.

Thirdly, we observed that the upregulated PPP2R2A is enriched in the PI3K/AKT pathway. It was reported that the activation of the PI3K/AKT pathway signaling contributes to lymphocyte-hyperactivation (55). PPP2R2A, as a regulatory subunit of PP2A, promotes the differentiation of Th1 and Th17 cells by activating the GEF-H1/RhoA/ROCK signaling pathway (56). Inflammation positively regulates Th17 differentiation through the phosphatase PP2A (57). In addition, PPP2R2A may also enhancing MAPK signaling through RAF and KSR or inhibit the MAPK signaling by regulating ERK (58). Furthermore, both PI3K and MAPK promote the production of type I IFN by regulating the nuclear translocation of IRF7 in human pDC (59). Therefore, upregulated gene PPP2R2A may play an important role on the pathogenesis of LN by enhancing lymphocyte hyperactivation and type I IFN-production.

The hemoglobin subunit beta (HBB) gene, which is expressed on erythroid lineage cells and involved in folate metabolism and innate immune response, was significantly upregulated in PBMCs, glomeruli, and renal tubules of LN patients (60). Similarly, elevated levels of HBB protein are reported in the serum of SLE patients (61) and in the urine of LN patient (62) compared to SLE without LN patients and healthy controls. Afridi et al. found that HBB polymorphisms upregulated the production of anti-malarial IgG (63). In addition, Wu et al. identified HBB as a major player in the iron death signaling pathway in diabetes (64). Sharma et al. also found HBB to be enriched in the iron homeostasis and iron death signaling pathways (65). Therefore, we speculate that HBB may be involved in the inflammatory process of LN through the regulation of IgG and iron death.

Finally, we observed a significant upregulation of TNFRSF17 in the PBMCs of LN patients compared to both control and SLE without LN groups. TNFRSF17 protein was highly expressed in the kidneys of LN patients, with distribution in the glomeruli, tubules, and interstitium, which supports the results of gene-level upregulation in both glomeruli and tubules observed in microarray analysis. Therefore, we believe that both gene and protein levels of TNFRSF17 are elevated in LN patients. In the current study, pathological classification of the involved LN patients included mainly III, IV, and V types,. Similar studies found that TNFRSF17 expression is significantly increased in the interstitium of proliferative LN patients (66), and in the glomeruli of V type LN patients (67). Our data support TNFRSF17 expression in class-switched memory B cells. TNFRSF17 mediates plasma cell survival through the classical NF-κB pathway, and may be a therapeutic target for plasma cells (6). We found that TNFRSF17 was significantly positively correlated with 24-hour urine protein quantification, and significantly negatively correlated with complement C3 and C4 levels. Studies revealed that the copy number variation (CNV) of the C4 gene and its related polymorphisms are associated with the susceptibility to SLE (68, 69). Specifically, low gene copy numbers (GCNs) of total C4 and the deficiencies of C4A were identified as medium to large effect size risk factors, while high copy numbers of total C4 or C4A were prevalent protective factors for European and East-Asian SLE patients (70). Complement C4 promote urine protein formation and LN development, and are negatively correlated with the severity of SLE (71). Therefore, we speculate that TNFRSF17 plays a pathogenic role in both PBMCs and local kidney tissue of LN patients, and further investigation into the possibility of targeting TNFRSF17 for the treatment of lupus is warranted.

The use of chimeric antigen receptor T cells (CAR-T) in autoimmune diseases has gained attention. Ellebrecht et al. designed a CAR targeting self-antigens to guide T cells in eliminating autoreactive B cells in pemphigus vulgaris (72). Mackensen et al. used anti-CD19 CAR-T cells to treat refractory SLE and found good patient tolerance. However, CAR-T cell therapy still faces challenges such as high cost, difficulty in production, the need for specific biomarkers to measure treatment efficacy, and safety concerns (73, 74). Therefore, we aimed to find a safe, specific, and efficient immunotherapy for the treatment of LN. The universal bispecific T cell engager IBI379 targets TNFRSF17 and CD3 in an asymmetric IgG-like format. In multiple myeloma (MM) studies, IBI379 effectively linked T cells and plasma cells, inducing T cell activation, proliferation, and clearance of TNFRSF17^+^ plasma cells (35). As IBI379 cannot bind to the TNFRSF17 of mouse, we conducted experiments by adding IBI379 to the culture of patients’ PBMCs. The results revealed that IBI379 effectively eliminated TNFRSF17^+^ plasma cells *in vitro*. However, its impact on B cells was not significantly different from the control group. These findings demonstrate the fast-acting and highly specific nature of IBI379, as it selectively targets pathogenic plasma cells expressing TNFRSF17 while preserving the patient’s B cell immune defense against infections, thus minimizing the risk of severe adverse reactions. Therefore, further *in vivo* studies are urgently needed to confirm the efficacy and safety of IBI379 as a potential therapeutic agent for LN.

To summarize, our study has identified a set of 70 unique genes specific to LN. These genes are enriched in the biological process class term (‘regulation of biological quality’) and the cell cycle pathway. Notably, the upregulation of SCARB2 presents a potential therapeutic target for inhibiting type I IFN production, while the downregulation of BUB1 may contribute to immune imbalance in LN. Furthermore, the upregulation of PPP2R2A may enhance lymphocyte hyperactivation and IFN production, while the upregulation of HBB may play a role in IgG production, iron death, and contribute to LN pathogenesis. Additionally, TNFRSF17 was found to be significantly upregulated in both PBMCs and kidney tissue of LN patients, and its expression level positively correlated with the levels of 24-hour urine protein. TNFRSF17 may promote plasma cell survival and contribute to LN pathogenesis, and the targeted drug IBI379 effectively induces apoptosis in plasma cells without affecting B cells in LN patients.

## Data availability statement

The datasets presented in this study can be found in the GEO repository (accession number: GSE211700). https://www.ncbi.nlm.nih.gov/geo/query/acc.cgi?acc=gse211700.

## Ethics statement

The studies involving humans were approved by Institutional Medical Ethics Review Board of the First Hospital of Jilin University. The studies were conducted in accordance with the local legislation and institutional requirements. Written informed consent for participation in this study was provided by the participants’ legal guardians/next of kin.

## Author contributions

XZ: Data curation, Investigation, Writing – original draft. MY: Funding acquisition, Validation, Writing – original draft, Investigation. ZY: Writing – review & editing, Investigation. TL: Investigation, Writing – review & editing. ZJ: Data curation, Funding acquisition, Writing – review & editing. YX: Writing – review & editing, Validation. ST: Writing – review & editing, Visualization. YL: Writing – review & editing, Validation. XW: Funding acquisition, Project administration, Supervision, Writing – review & editing.

## Supplementary material

Supplementary Table 1 Clinical information of the high-throughput sequencing samples.

Supplementary Table 2 The top 20 upregulated genes in LN compared to control.

Supplementary Table 3 The top 20 downregulated genes in LN compared to control.

Supplementary Table 4 The top 20 upregulated genes in LN compared to SLE without LN.

Supplementary Table 5 The top 20 downregulated genes in LN compared to SLE without LN.

Supplementary Table 6 Clinical information of the samples for qRT-PCR and cell culture.

Supplementary Table 7 Primers used in qRT-PCR.

Supplementary Table 8 Clinical information of the samples for immunohistochemistry.

Supplementary Figure 1 Gene expression and cell distribution in the single cell sequencing of human kidney. (Single Cell Expression Atlas, Spatio-temporal immune zonation of the human kidney, https://www.ebi.ac.uk/gxa/sc/experiments/E-HCAD-10/results/tsne).

Supplementary Figure 2 Gene expression and cell distribution in the single cell sequencing of mouse kidney. (Single Cell Expression Atlas, Single cell RNA sequencing analysis of mouse resident and infiltrating immune cells during acute kidney injury and fibrosis, https://www.ebi.ac.uk/gxa/sc/experiments/E-MTAB-8002/results/tsne).

Supplementary Figure 3 Histogram illustrating the gene expression levels in peripheral blood mononuclear cells (PBMCs) of healthy human subjects, as determined by single-cell sequencing data. The data source for this analysis is available from EMBL’s European Bioinformatics Institute (website: www.ebi.ac.uk/gxa/experiments/E-MTAB-3827/Downloads?specific=true&geneQuery=%255B%257B%2522value%2522%253A%2522ensg00000106799%2522%257D%255D&filterFactors=%257B%257D&cutoff=%257B%2522value%2522%253A0.5%257D&unit=%2522FPKM%2522).

Supplementary Figure 4 Correlation analysis between the percentage of TNFRSF17+ area and AI **(A)** or CI **(B)**.
