# Supplementary figures and images for: Uncovering lupus nephritis-specific genes and the potential of TNFRSF17-targeted immunotherapy: a high-throughput sequencing study
Source: Front Immunol. 2024 Feb 19;15:1303611. doi: 10.3389/fimmu.2024.1303611 (PMC10909935; doi:10.3389/fimmu.2024.1303611)

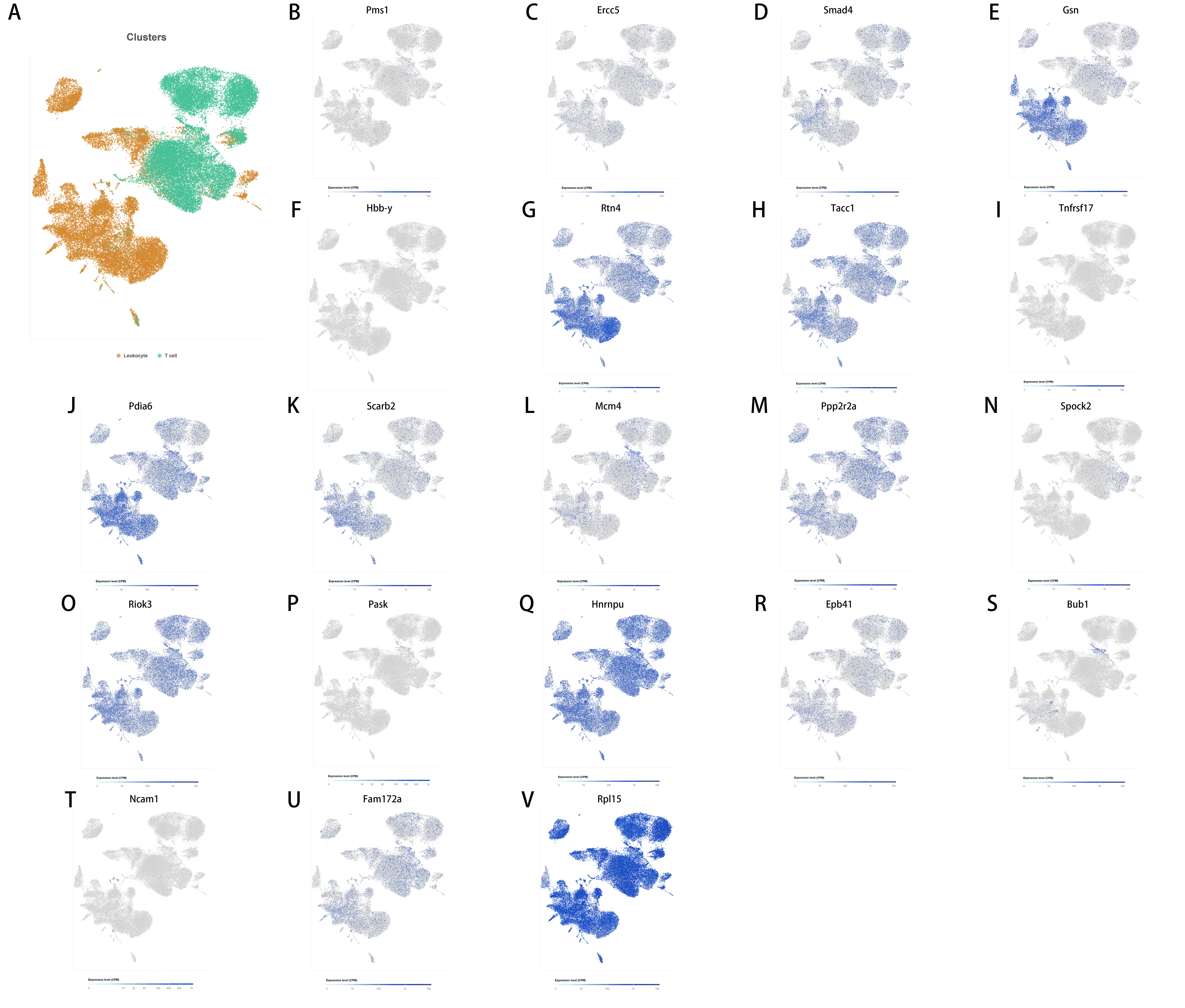

Supplement: Supplementary Figure 2 — Gene expression and cell distribution in the single cell sequencing of mouse kidney. (Single Cell Expression Atlas, Single cell RNA sequencing analysis of mouse resident and infiltrating immune cells during acute kidney injury and fibrosis, https://www.ebi.ac.uk/gxa/sc/experiments/E-MTAB-8002/results/tsne). [file Image_2.jpeg]

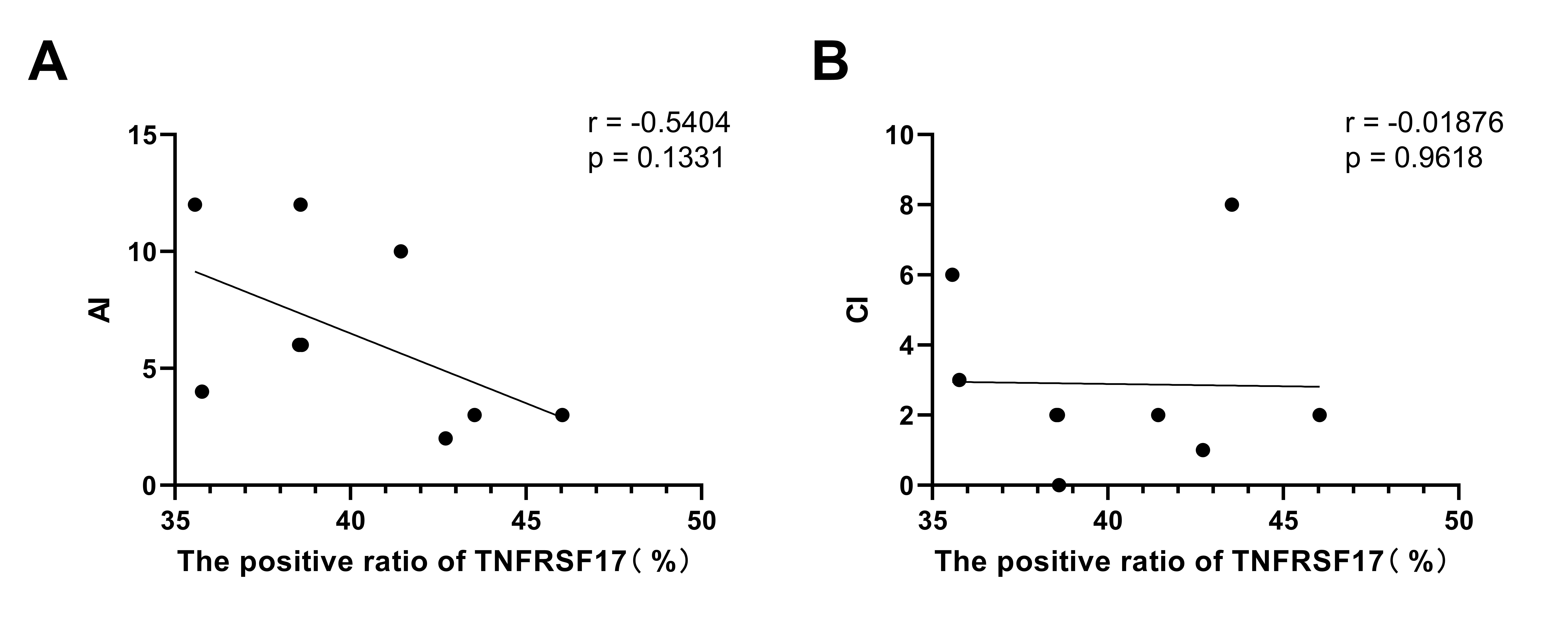

Supplement: Supplementary Figure 4 — Correlation analysis between the percentage of TNFRSF17+ area and AI (A) or CI (B). [file Image_4.jpeg]
